# New insights into phenotype and genotype relationships in *Neospora caninum*

**DOI:** 10.3389/fvets.2023.1214971

**Published:** 2023-08-17

**Authors:** Andres Cabrera, Luisa Berná, Lucía López, Paula Faral-Tello, Ana Paula Arevalo, Martina Crispo, Maria E. Francia, Carlos Robello

**Affiliations:** ^1^Laboratorio de Interacciones Hospedero-Patógeno -UBM, Institut Pasteur de Montevideo, Montevideo, Uruguay; ^2^Departamento de Parasitología y Micología, Facultad de Medicina, Universidad de la República, Montevideo, Uruguay; ^3^Laboratorio de Genómica Evolutiva, Facultad de Ciencias, Universidad de la República, Montevideo, Uruguay; ^4^Laboratory Animals Biotechnology Unit, Institut Pasteur de Montevideo, Montevideo, Uruguay; ^5^Laboratory of Apicomplexan Biology, Institut Pasteur de Montevideo, Montevideo, Uruguay; ^6^Departamento de Bioquímica, Facultad de Medicina, Universidad de la República, Montevideo, Uruguay

**Keywords:** *Neospora caninum*, virulence, chronicity, vertical transmission, abortion, animal health, single nucleotide polymorphisms

## Abstract

The successful isolation of four new *Neospora caninum* strains from different regions and with different backgrounds (obtained from an abortion storm or congenitally infected and asymptomatic calves) allowed us previously to characterize natural isolates, finding differences in phenotype and microsatellites. Given the variability observed, we wondered in this work whether these differences had consequences in virulence, invasion and vertical transmission using cell cultures and murine neosporosis models. In addition, we performed the genomic analysis and SNP comparative studies of the NcURU isolates. The results obtained in this work allowed us to establish that NcURU isolates are of low virulence and have unique phenotypic characteristics. Likewise, sequencing their genomes has allowed us to delve into the genetic singularities underlying these phenotypes, as well as the common mutated genes. This work opens a new perspective for diagnostic purposes and formulating possible vaccines based on attenuated strains.

## Introduction

*Neospora caninum* is a coccidian parasite of the Apicomplexan phylum, and the causative agent of bovine and canine neosporosis (1). In domestic and wild dogs, neosporosis may be a debilitating neurological disease. In addition, Bovine neosporosis is one of the leading causes of bovine abortion worldwide. Due to the burden on the economy worldwide, neosporosis is known as the billion-dollar disease (2). As part of its obligatory intracellular life cycle, *N. caninum* actively invades the cells of its hosts (1). Acute infections caused by the rapidly dividing *N. caninum* tachyzoites can evolve into chronically persistent, slow-growing bradyzoites (3). Bradyzoites are protected from the host’s immune response by encysting in immune-privileged tissues, such as the brain (3). These latent forms can reactivate during pregnancy and efficiently transmit from an infected mother to the fetus (4). Drugs to efficiently treat the chronic form of the disease are unavailable, and vaccination is considered the best potential control strategy against bovine neosporosis, as it offers the best cost–benefit ratio (5).

Molecular studies over the past decade have reported the occurrence of phenotypic variability in different naturally occurring *N. caninum* strains isolated worldwide (6). This phenotypic variability is accompanied by genetic variability, identified by the analysis of microsatellites and single nuclear polymorphisms (7–10). However, our grasp on the extent of genetic variability among strains of *N. caninum* and its correlation with phenotypic variability is still insufficient (11). This is partly due to the relatively low number of genetic/phenotypically characterized natural isolates pursued worldwide. In this context, the continued isolation of wild parasite strains exhibiting marked differences in virulence and identifying the genetic basis of these phenotypes is crucial to further our understanding of the molecular basis of pathogenesis in these parasites.

The murine model has been utilized for many years to isolate and characterize *Neospora caninum* strains (11). Despite being a well-studied model that enhances our understanding of neosporosis, including infection, immune response, vertical transmission, and other parameters, it continues to be a valuable approach for replicating the bovine model (12–16).

In previous work, we isolated four strains of *N. caninum* (7). We determined that these four strains represent three distinct genetic lineages based on microsatellite typing, differing significantly from other extensively characterized isolates, such as the reference strain *N. caninum* Liverpool (NcLiv). We also observed that some lineages were more closely related to abortion-causing strains than others foretelling possible phenotypical differences among isolates. With these results in mind, we set out to characterize the phenotypes of isolates NcUru1 thru 4 and their correlation with genetic variability, as determined by whole-genome sequencing.

## Materials and methods

### Cell culture and purification of tachyzoites

*Neospora caninum* Liverpool was acquired from ATCC (Cat.#: 50845). NcUru1-4 were isolated from local congenitally infected calves and functionally and genetically characterized (7). All strains were cultured and maintained by serial passages in human foreskin fibroblasts (HFF, ATCC #SCRC 1041) in DMEM (Gibco™, Thermo Fisher Scientific, Waltham, MA, United States), supplemented with 10% Fetal Bovine Serum (Invitrogen, Carlsbad, CA, United States), 1% penicillin/streptomycin (Invitrogen, Carlsbad, CA, United States). Cells were incubated at 37°C and 5% CO_2_. The infected HFF monolayers were removed with a cell scraper, centrifuged and washed three times with PBS at 500x g for 10 min. Subsequently, the parasites were resuspended in PBS, passed three times through an 18G needle, and filtered through a 3-uM pore-size filter. The number of tachyzoites was determined by using a Neubauer chamber.

### Western blotting and immunofluorescence assays

For immunoblots protein extracts of *N. caninum* tachyzoites were analyzed as previously described (17). Briefly, total parasite proteins (30 ug per well) were separated on 12% SDS-polyacrylamide gels, and bands were visualized by Coomassie Blue staining or transferred to polyvinylidene fluoride (PVDF) membranes (Millipore, MA, United States) together with a visible prestained protein marker (PageRuler™ Prestained Protein Ladder, Thermo Scientific™). The membranes were blocked with 5% (w/v) skim milk in PBS for 1 h at 37°C, rinsed with a washing buffer, and incubated with mouse serum (1:100) overnight at 4°C. The blots were washed five times with PBS-T (1% Tween-20), followed by incubation with horseradish peroxidase (HRP)-labeled goat anti-mouse IgG (H + L) (1:5,000, Sigma, United States). Finally, enhanced chemiluminescence reagents (SuperSignal™ West Pico PLUS Chemiluminescent Substrate, Thermo Scientific™) were used to observe the reaction bands after 30 s exposure time.

Immunofluorescence assay of intracellular tachyzoites grown on HFFs was performed using a commercial *N. caninum* antiserum raised in goats (1:100; VMRD, Pullman, WA, United States). Donkey anti-goat IgG H&L-FITC conjugated (Abcam, Cambridge, United Kingdom) was used at a 1:1000 dilution as the secondary antibody. The acquisition was carried out in an epifluorescence microscope (Olympus Life Science, Tokyo, Japan) using a × 100 oil objective (7).

### *In vivo* studies in mice

For *in vivo* studies, 6–8 week-old BALB/cJ mice (Jax# 000651) were used following approved institutional animal care and use protocols. Mice were bred at the animal facility of the Laboratory Animals Biotechnology Unit of Institut Pasteur de Montevideo under specific pathogen-free conditions in individually ventilated racks (IVC, 1285 L, Tecniplast, Milan, Italy). The housing environmental conditions during the experiment were as follows: 20 ± 1°C temperature, 30–70% relative humidity, negative pressure (biocontainment), and a light/dark cycle of 14/10 h. All procedures were performed under Biosafety level II conditions. Mice were housed in groups of five for each of the six conditions (PBS, NcLiv and NcURU 1–4), and the whole experiment was performed by duplicate, meaning that 10 mice were used per each condition.

Parasites in tissue culture were mechanically extracted from the host cells by repeatedly passing them through a 26G syringe, filtered through a 3 μm pore size polycarbonate filter, and counted in a Neubauer chamber (7). To prevent the selection of *in vitro* culture phenotypes, all strains used in phenotypic and *in vitro* assays were passed less than 12 times from the time of isolation to when the assays were performed.

Beforehand, the LD_50_ was determined for our commercial *NCLiv* strain in Balb/cJ female mice. The estimated LD50 was 1×10^7^ Liverpool tachyzoites inoculated intraperitoneally.

This dose (10^7^) was used in all assays to determine strain-specific virulence. Chronic disease was induced in 6–8 week old female BALB/cJ mice by intraperitoneal inoculation of a sublethal dose of 10^6^ tachyzoites, as determined previously based on the literature (13). Mice were visually inspected daily for 30 days post-inoculation to detect clinical signs compatible with neosporosis (rough fur, inactivity, anorexia and neurological signs). Seropositivity against *N. caninum* infection was systematically tested two weeks post-infection *via* submandibular bleeding. Sera were tested by immunoblotting against *N. caninum* tachyzoites lysates. Mice that tested positive for ELISA were euthanised using humane methods, and aseptic brain samples were collected for subsequent analysis. PCR was performed using the previously described primers NP6 (5′-CAGTCAACCTACGTCTTC-3′) and NP21 (5’-GTGCGTCCAATCCTGTAA-3′) (18) to detect parasite DNA in the brain samples. Concurrent positivity in both serology and PCR was considered indicative of a chronic infection.

### Vertical transmission assays

For vertical transmission studies, chronically infected or a control group of non-infected female mice were housed in groups of six for each of the six conditions (PBS, NcLiv and NcURU 1–4) and the experiment was performed by triplicate, meaning that 18 mice were used per each condition.

Ten-week-old mice were synchronized in their estrous by exposing them to male dirty bedding, as previously described (19). After estrous synchronization, three females were placed with a single male for seven nights. The presence of a vaginal plug was corroborated daily in all cases as a positive sign of mating and considered day 0 of pregnancy. Pregnant mice and fetuses were humanely euthanized at day 20 post-coitum, and brain DNA from mothers and viable fetuses was extracted for PCR assays. Sera were collected from the submandibular vein of BALB/cJ mothers for serological assays.

### Invasion and intracellular growth assays

For the intracellular growth assay, confluent monolayers of human foreskin fibroblasts (HFFs) were grown on coverslips and subsequently inoculated with 10^3^ freshly released tachyzoites. Two hours post-inoculation, cells were washed five times with PBS to remove unattached parasites and fixed with 4% paraformaldehyde 24 h later. Samples were analyzed by immunofluorescence assays, as described by Cabrera et al. (7). The number of parasites per vacuole in 200 vacuoles per coverslip was determined in triplicate. Confluent HFFs grown on coverslips were kept on ice before the invasion assays. Upon inoculation with 1,000 parasites, plates were centrifuged at 300 × g for 10 min at 4°C to ensure all strains contacted the cells simultaneously. Plates were then switched to 37°C for 30 min, washed with PBS three times to remove unattached parasites, and fixed with 4% paraformaldehyde. The number of parasites per field in 50 randomly selected fields was determined for all strains, in triplicate, by immunofluorescence assay.

### Sequencing, alignment, and variant calling

DNA was extracted using a DNA purification kit from Zymo Research (#D4074). Illumina libraries for *N. caninum* NcUru1, NcUru2, NcUru3 and NcUru 4 were performed as described (20), and whole genome sequencing of the strains was performed with 2×100 paired-end cartridges. Reads were mapped to the new reference *N. caninum* Liverpool genome (21) using Burrows-Wheeler Aligner (BWA 0.7.17) (22) mem using default parameters. Samtools v1.9 was used to post-process alignment files (22). SNP and Indel discovery was performed with Samtools (23) and VarScan. v2.3.9 (24) with parameters –min-reads2 3 –min-coverage 3 –min-var-freq 0.8. SNPeff v.5.0 was used to annotate genomic variants and predict their functional effects. IGV was used for alignment and variant visualization (25). Venny 2.1 was used for Venn diagram plots (26). For the NcUru4 isolate the sequencing coverage was not enough for this analysis.

### Ethics statement

All animal experiments were done following international animal care and use guidelines, as well as respecting National Law 18.611 with pre-approved protocols by Institutional Ethics Committee (CEUA # 010–17) at the Institut Pasteur de Montevideo.

## Results

### NcUru isolates exhibit comparatively lower virulence than NcLiv

**Figure 1:**
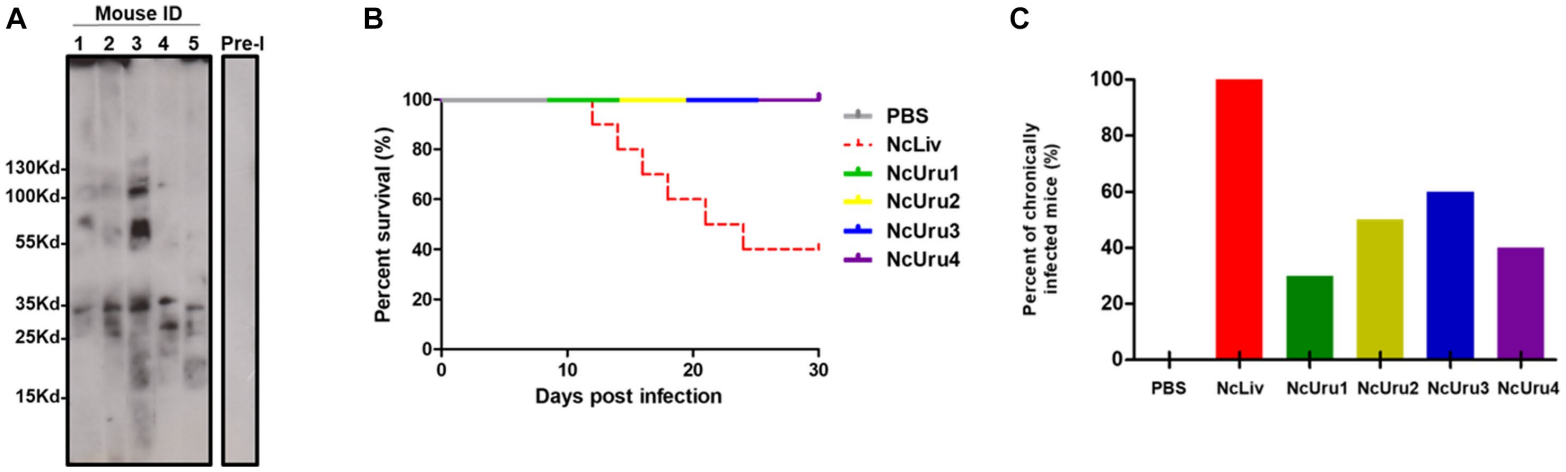
NcUru 1–4 isolates exhibit comparatively lower virulence and a tendency to evolve into a chronic infection than *NcLiv.* **(A)** Representative Western blot showing seroconversion of infected mice with NcUru1. Sera from mice 1 thru 5 were assayed two weeks post-inoculation. Pre-immune sera were pooled and are shown as a control of seronegativity before infection. **(B)** Survival rate of parasite-infected mice inoculated with 10^7^ tachyzoites of the indicated strain or PBS (negative control). **(C)** Percentage of positively detected infected brains by PCR in all mice inoculated with 10^7^ parasites of the indicated strain (both surviving and succumbing to the infection).

To comparatively assess the phenotypes of NcUru1-4, we first evaluated there *in vivo* virulence. To do this, we infected immunocompetent Balb/cj mice with 10^7^ freshly purified parasites of each of the four isolates. NcLiv was used as positive and PBS as negative controls. Mice seroconversion was determined by Western blot two weeks after inoculation, considering as positive the presence of the expected discrete bands of 17 kDa, 29–37 kDa and higher than 70 kDa (27, 28) (Figure 1A). Clinical signs of the disease (anorexia, rough coat, and inactivity followed by nervous signs like rounded back, pelvic limb weakness and walking in circles) were monitored for a month post-inoculation.

As expected, 60% of the mice infected with the reference strain NcLiv had to be euthanised due to the infection within a week. In contrast, mice infected with NcUru1-4 showed no obvious clinical signs of the disease and exhibited equal survival as that of the PBS control (Figure 1B). In addition, all strains were detectable by PCR in the brains of the surviving mice, which suggests that they were all able to transition from the acute to the chronic form. However, while a positive PCR was detectable in all the brains of mice infected with NcLiv (both surviving and euthanized due to the infection), NcUru1-4 were consistently detectable in 30, 50, 60 and 40% of mice, respectively. This suggests that the ability to persist in the host chronically varies significantly with respect to *NcLiv*, but also among NcUru strains (Figure 1C). However, all mice, both surviving and euthanized, seroconverted two weeks post-inoculation (shown only for five mice infected with NcUru1 in Figure 1A).

### Infection with autochthonous and reference strains do not generate changes in the pregnancy rate

**Figure 2:**
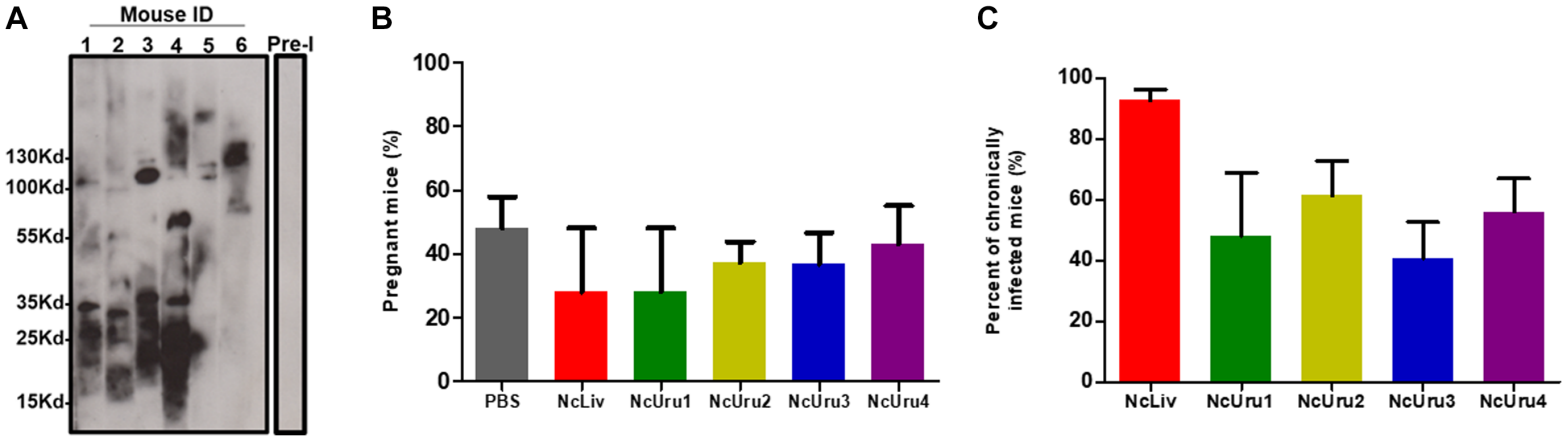
Infection with autochthonous and reference strains does not generate changes in the pregnancy rate. **(A)** Representative Western blot showing the seroconversion of six female mice two weeks following inoculation with a sub-lethal dose of NcUru1 (1–6). Preimmune serum was pooled and assayed before infection and is shown as a control (7). **(B)** Percentage of pregnant mice inoculated with the strains indicated. **(C)** Percentage of positively detected infected brains by PCR in all mice inoculated with a sublethal dose (106) of the indicated strains as determined post-mortem. Data are shown as the mean and SEM of 3 independent experiments.

We next evaluated the strain-specific impact on pregnancy outcomes. To do this, we optimized a murine model of vertical transmission. In short, we infected 6-8-week-old BALB/cJ female mice with 10^6^ parasites in order to generate subclinical chronic infection. We next crossed females and males, whereby subgroups of females infected with the same parasite strain were crossed with the same male. Every experiment was done in triplicate using six female mice per parasite strain per experiment. We note again that all mice effectively seroconverted two weeks after inoculation (Figure 2A). Pregnancy rates (pregnant/mated females) did not show significant differences. However, they varied from 28% for females inoculated with *NcLiv* (positive control), to 48% for the control group (PBS) (Figure 2B). In the case of NcLiv, chronic infection of the mother was corroborated postmortem in all cases. The *NcUru* isolates impacted pregnancy rates differently but within the ranges of the positive and negative controls. Pregnancy rates in females infected with NcUru1 were comparable to that of NcLiv (28%). NcUru4 behaved similarly to the negative control (PBS- 48%) (Figure 2B).

Chronic infection of the females was evaluated postmortem by PCR. The results were comparable to those of the higher inoculum infection described above. These results reinforce that the observable differences in the establishment of chronic infection are intrinsic strain-specific characteristics (Figure 2C). Pregnancy, abortion and the vertical transmission rate for each strain were recorded.

### The NcUru isolates exhibit lower vertical transmission rates than the reference strain

**Figure 3:**
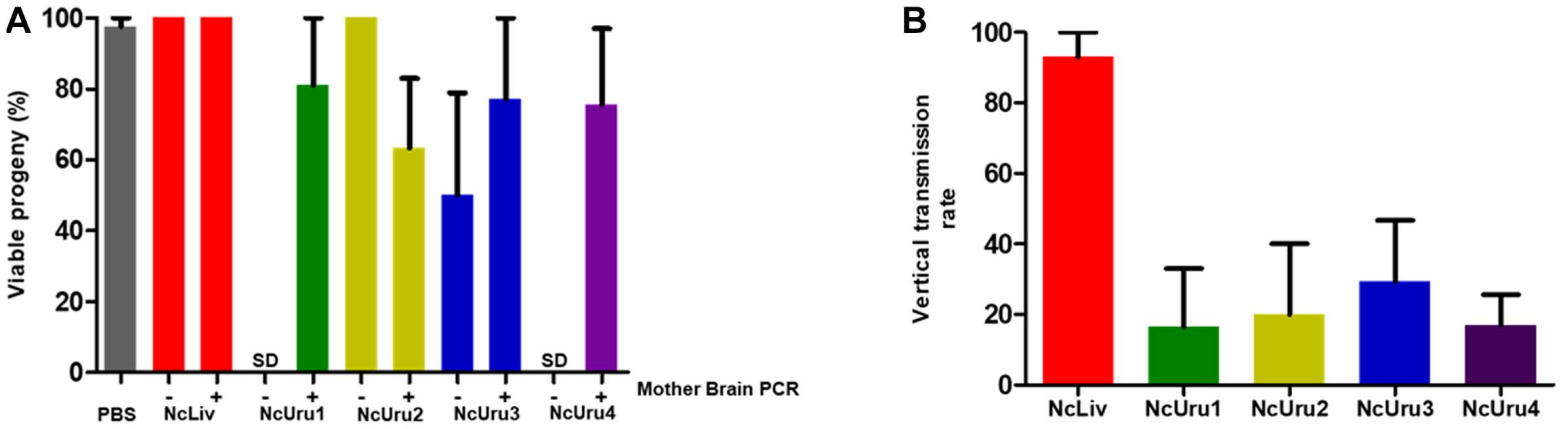
The NcUru isolates exhibit lower vertical transmission rates than the reference strain. **(A)** Graphical representation of viable progeny percentages obtained following infection with the indicated strains. Mother brains were evaluated by PCR post-mortem for the presence of parasite DNA to determine chronicity (+), absence of parasite’ DNA (−), or No Data (SD). **(B)** Graphical representation of the percentage of infected progeny as determined by PCR detection of parasite DNA in the brains of viable fetuses.

Mice were euthanized a day before the expected due date. This allowed us to correlate the impact of given mouse chronicity on the offspring. We observed that virtually all offspring were viable in both negative (PBS) and positive control (NcLiv) groups; 98 and 100%, respectively (Figure 3A). When the progeny of mice infected with NcUru isolates were evaluated, we observed that the output of viable offspring significantly decreased only for mothers displaying a chronic infection with NcUru2. Chronically infected mice with NcUru2 bear only 63% of viable offspring. In contrast, mice inoculated with NcUru2 who did not develop a chronic infection had 100% viable offspring, suggesting that the impact on offspring viability is directly related to the chronic presence of NcUru2. NcUru3-chronically infected mothers yielded 77% viable pups, while those chronically infected with NcUru4 exhibited 75% viable offspring. Interestingly, progeny viability for the NcUru3 was still halved in non-chronically infected mothers, suggesting that both previously acquired acute infections and chronic persistence of this strain have a sizable impact on pregnancy rates (Figure 3A). NcUru1-infected mothers yielded an average of 81% viable offspring (Figure 3A).

To compare strain-specific vertical transmission rates, we determined the percentage of *N. caninum* positive fetuses by PCR detection of the parasite DNA in the brains of viable offspring (Figure 3B). Consistent with previous reports, we detected a vertical transmission rate of 93% in three independent experiments for NcLiv. Vertical transmission rates for NcUru1 thru 4 were comparatively lower than that of NcLiv. NcUru2 was detectable only in 20% of the progeny, while the vertical transmission rate was 29 and 17% for mice infected with NcUru3 and NcUru4, respectively. Remarkably, NcUru1 was only detectable in 16% of the progeny (Figure 3B). The latter is consistent with NcUru1-infected mothers of all strains, yielding the highest percentages of viable offspring. We note that NcUru1-infected mothers did not get pregnant for the first replicate experiment (see Table 1).

**Table 1:** Statistics of whole genome sequencing of three *N. caninum* Uruguayan strains.

Strain	Aligned reads	Aligned reads (%)	Coverage (%)	Total SNPs	Total SNPs in coding genes
NcUru1	4.11E + 06	9.5	6.8	638	531
NcUru2	8.19E + 06	15.0	13.5	1,436	1,053
NcUru3	3.90E + 06	6.1	6.4	1,175	871

### Invasion and intracellular growth of isolated strains

**Figure 4:**
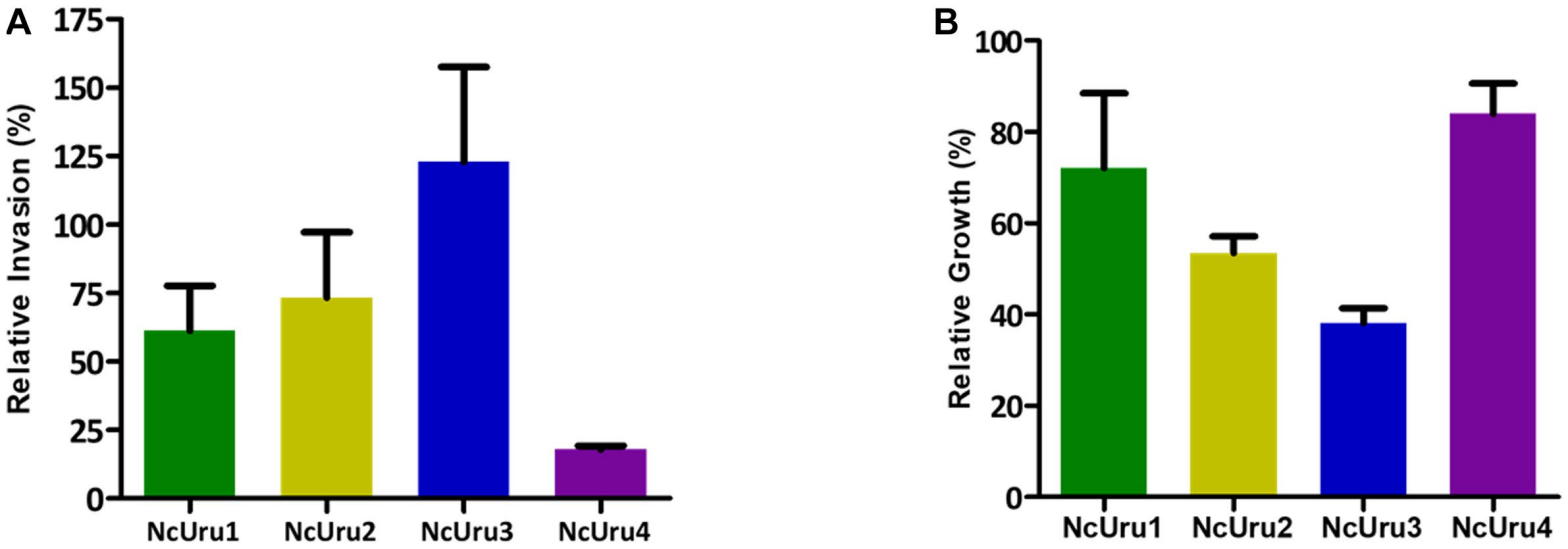
Invasion and intracellular growth of isolated strains were quantified and plotted in comparison to the reference strain. **(A)** Relative invasion rate of each strain was quantified against that of NcLiv (*n* = 3, Average, SEM are plotted). **(B)** Relative intracellular growth of each strain was quantified against that of NcLiv (*n* = 3, Average, SEM are plotted).

It is well established for *N. caninum*’s close relative, *Toxoplasma gondii,* that the capacity to invade cells and velocity of intracellular growth correlates with virulence (29). To approach the molecular basis of the phenotypic differences observed *in vivo*, we *in vitro* characterized each strain’s ability to invade a host cell and their intracellular growth rate using NcLiv as a reference. These assays revealed that NcUru1 is half as invasive as NcLiv, while NcUru2 invades roughly 25% fewer cells than NcLiv in the same timeframe. NcUru3 invades host cells with an efficiency similar to the reference (Figure 4A). Remarkably, NcUru4 exhibits a significantly reduced invasion rate (18%) (Figure 4A). Conversely, when the relative intracellular growth of each strain was evaluated, we found that NcUru4 exhibits a similar growth rate to that of NcLiv, suggesting that those parasites which manage to invade are equally able to grow intracellularly. NcUru1 (72%), NcUru2 (53%) and NcUru3 (38%) were comparatively slower growing than NcLiv (Figure 4B). The reduced efficiency of NcUru1 in invasion and intracellular growth are in consonance with its reduced efficiency in generating chronic infection and lower vertical transmission rates.

### SNP calling and comparative analyses of isolates

**Figure 5:**
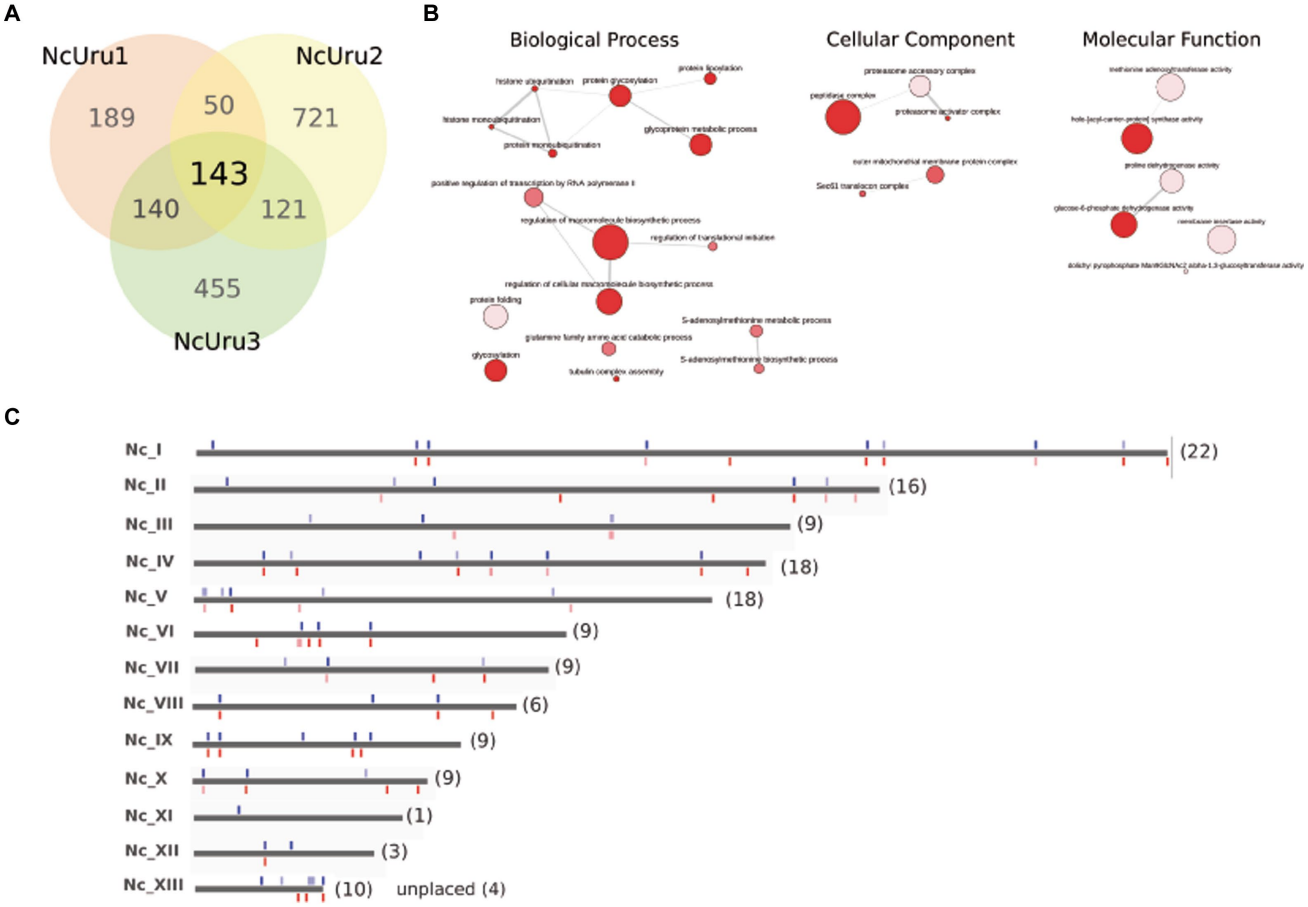
SNPs/Indels differences among NcURU strains. **(A)** Venn diagram. **(B)** Gene ontology analysis of the 143 common genes. **(C)** Chromosome localization of the 143 common SNPs.

To determine the mutations responsible for observed phenotypical differences, we sequenced the complete genomes of NcUru isolates. A large number of genetic variations (2591) were identified among the different strains, including heterozygous SNPs/Indels. Of these, 1819 were located in coding regions (Supplementary Table 1). We identified the genes affected by strain-specific SNPs in our newly annotated genome (21). Notably, most of the mutations were strain-specific, with 189 found in NcUru1, 721 in NcUru2 and 455 in NcUru3. Only 143 mutations were common to all three NcUru isolates (Supplementary Table 2), and 35% corresponded to hypothetical proteins (Supplementary Table 3). Of the annotated genes, 9% contain functional domains related to surface proteins (SRS domains), while the rest mainly correspond to translation-related genes (ribosome biogenesis, ribosomal proteins and elongation factors), protein degradation (ubiquitin proteases and proteasome subunits), AP2 transcription factors and regulatory proteins, including mainly kinases and phosphatases (Supplementary Table 3). The 143 shared genes are enriched in GO terms related to pentose-phosphate pathways and proteasome/ubiquitination (Figure 5), whereas GO terms of strain-specific genes show the predominance of pentose phosphate pathway and cation binding related genes in NcUru1, lipid metabolism and membrane traffic in NcUru3. No significant GO terms were found in NcUru2 (Supplementary Table 1).

## Discussion

In previous work, we isolated and genetically typed four *N. caninum* strains named NcUru1 thru 4 (7). We further showed that while NcUru1, 3 and 4 grouped genetically, they were markedly distinct from both NcUru2 and NcLiv, the latter being the strain commonly used as a reference for the study of *N. caninum*’s biology. In this work, we have delved into the study of these natural isolates by characterizing strain-specific phenotypes relevant to the clinical outcomes of neosporosis. All the natural isolates exhibited low virulence (100% survival) compared to NcLiv, the most widely used reference strain, which is highly virulent. However, all of the strains triggered seroconversion, as evidenced by immunoblots. In all positive sera, bands corresponding to the most immunodominant antigens were present. Although heterogeneous depending on the isolate, these bands have been described as discrete, with estimated MW of around 17 kDa, 29–37 kDa and higher than 70 kDa (27, 28). Mice were able to reach the chronic phase, although at different rates. Notably, NcUru1 exhibited a markedly reduced tendency to generate chronic infection. These results are consistent with observations reported by others whereby low virulence strains do not elicit clinical disease or establish chronic infection in mice (30, 31). It is important to mention that NcLiv has been a laboratory-adapted strain for decades, while NcUru isolates have been recently obtained, which leads to reconsidering the limits of high virulence reference strains, although virulent strains can be attenuated through several culture passages (32). Subtle phenotypic differences were identified among all strains, especially in their behavior in pregnancy- arguably the most relevant epidemiological setting for this infection. We observed that, in general, pregnancy rates were not influenced by the infection with *N. caninum.* Remarkably, in the case of mice inoculated with NcUru1 and NcUru4, none of the non-chronically infected mice got pregnant. Though these phenomena could be unrelated, their relation to immune responses deserves to be studied, although it may also be related to the low rates of reactivation of the infection of the strains (33). The NcLiv vertical transmission rate (87%) observed in this work is comparable with previous studies confirming that this strain is very effectively transmitted by an infected mother while causing little to no abortions (30, 31, 34). The NcUru isolates exhibited a very low vertical transmission rate compared to the reference strains, with NcUru1 being passed on to less than one every five progeny. Differences in vertical transmission rate have also been observed between another virulent isolate (Nc-1) and a number of low-virulence strains isolated in Spain (35). The higher rates of vertical transmission *in vivo* do not necessarily indicate greater placental tropism in this strain but rather that it may be a consequence of its high virulence, while the NcUru isolates, despite their low virulence, still retain the ability to be passed on to offspring. In another study with the bovine placenta, different strategies were observed for this parasite depending on the virulence of the strain (36). It is worth mentioning that all of NcUru isolates were obtained from congenitally infected calves, implying that they are indeed transmitted vertically in the wild.

The *in vivo* and *in vitro* evaluation of NcUru1 thru 4 revealed, somewhat expectedly, marked phenotypic differences among strains. These findings are consistent with our previous work showing that these strains are genotypically distinct by microsatellite typing (7). Specifically, NcLiv is clustered in a markedly different genetic group from the NcUru strains. NcUru1 and 4 were genetically identical in their microsatellite profile, while NcUru3 clustered closely. NcUru2 groups with a distinct genetic cluster, suggesting it is genetically the most distanced strain of the group (7). The incongruence between phenotype and genotypes of isolates 1 and 4 can be explained by the fact that microsatellite analyses constitute an initial approach to determine genetic differences, but it does not constitute an in deep genomic characterization. To delve into genetic differences among NcUru strains that could underlie their distinct phenotypic traits, we pursued whole-genome sequencing for NcUru1-3. We repeatedly failed to obtain sufficiently good-quality DNA for sequencing NcUru4. We hypothesize that this was partly due to its reduced invasion efficiency, leading to an increased rate of uninfected host cells in tissue culture contributing towards total DNA when purified.

We previously re-sequenced and assembled the whole genome of NcLiv and NcUru1 using third-generation sequencing and showed that the *N. caninum* genome previously accepted as the reference had been assembled incorrectly. Furthermore, we demonstrated that the genomes of NcLiv and NcUru1 are virtually identical regarding gene composition, genome organization and synteny conservation, and no large rearrangements or deletions were detected (21). We now mapped the Illumina sequences of NcUru1-3 using our newly assembled NcLiv genome as the reference and identified their differences. The whole-genome sequencing of NcUru isolates revealed that while most non-coding mutations are strain-specific, shared variants are present mostly in coding regions. A total of 143 mutations were identified in the three NcUru strains evidencing, albeit expected, differences with NcLiv that could be characteristic of regional strains. These variants could be related to phenotypic differences as they affect genes involved in the different pathways that could conceivably modulate the host response. We highlight, among them, the SRS surface proteins, the outer mitochondrial membrane proteins and the proteasome. The SRS family corresponds to surface glycoproteins found only in Coccidia, which have been associated with invasion and immune response (37, 38). Mitochondrial proteins could participate in the modulation of the innate immune response (39, 40). The relevant role of Toxoplasma’s interaction with host mitochondria was recently demonstrated (41). Finally, changes at the proteasome level can affect both *N. caninum* and the host, as it has been evidenced in *Toxoplasma gondii* (42).

We observed that the SNPs we detected in our study did not align with hotspots previously described in other work. Calarco et al. (43) reported multiple SNP hotspots between two strains (Liverpool and Norwa) on chromosomes XI V, VI, XI, and XII (2012 version). More recently a study using seven additional Nc strains for whole genome sequencing revealed SNPs hotspots common to five strains, with most SNPs clustered in six discrete chromosomal regions (9). However, it is important to note that both studies used the previous NcLiv reference genome, which has incorrectly assembled and erroneously defined chromosomes to map these hotspots. In addition, many identified hotspots are not consistent between the two reports.

In our study, we did not observe the presence of discrete genomic regions in which SNPs were concentrated, nor did we preferentially detect SNPs in previously defined hotspots (44). Although there were some limitations in sequence coverage, which affected our ability to identify all variants confidently, there were also similar limitations in the work of Khan and colleagues, in which sequence coverage was relatively low for some strains. Notably, while our results do not support the presence of discrete genomic regions in which SNPs are concentrated, it is important to acknowledge that further investigation may be necessary to fully understand the existence and potential artifactual nature of hotspots described in previous studies.

In conclusion, despite their similarity in microsatellite patterns, we have shown ample biological diversity among NcUru isolates. In particular, we note that these isolates are generally of low virulence, particularly NcUru1 shows a reduced impact on detrimental pregnancy outcomes. Finally, our work constitutes proof of the importance of working on correctly assembled and thoroughly annotated genomes when analyzing the impact of genetics on observable phenotypes. We are currently working on deciphering the functional association between strain-specific non-synonymous gene mutations and their impact on virulence traits relevant to the overarching goal of developing a live attenuated vaccine.

We recognize the limitations of our work related to NcLiv, i.e., laboratory-adapted parasites, although this is a point to consider in most works using this strain. This point, in addition to the mouse model, means that it is not clear whether our results can be transferred to another animal species. Another limitation is that although the NcUru strains can be used as live attenuated vaccines, their isolation from congenitally infected calves carries the risk that they can be more easily vertically transmissible.

## Data availability statement

The datasets presented in this study have been deposited in NCBI as BioProject ID: PRJNA977643.

## Ethics statement

The animal study was approved by all animal experiments were done following international animal care and use guidelines, as well as respecting National Law 18.611 with pre-approved protocols by Institutional Ethics Committee (CEUA # 010–17) at the Institut Pasteur de Montevideo, URUGUAY. The study was conducted in accordance with the local legislation and institutional requirements.

## Author contributions

CR conceived the global project and obtained support. AC, MF, and CR contributed to the conception and design of the study and wrote the first draft of the manuscript. AC, LL, APA, and MC contributed to the study of animal models. LB analyzed the genomes and organized the database. MC and LB wrote sections of the manuscript. All authors contributed to the manuscript revision and read and approved the submitted version.

## Funding

This project was funded by grant FSSA_X_2014_ 1_106026 and FSSA_1_2019_1_160691 from the Uruguayan National Agency for Research and Innovation (ANII) awarded to CR. AC was supported by a doctoral fellowship from ANII. MF was supported by a Calmette and Yersin (Pasteur Network) and ANII postdoctoral fellowships. AC, LB, PFT, MF, MC, and CR are researchers from PEDECIBA (Programa de Desarrollo de las Ciencias Básicas, Uruguay) and the Sistema Nacional de Investigadores (SNI-ANII, Uruguay).

## Conflict of interest

The authors declare that the research was conducted in the absence of any commercial or financial relationships that could be construed as a potential conflict of interest.

## Publisher’s note

All claims expressed in this article are solely those of the authors and do not necessarily represent those of their affiliated organizations, or those of the publisher, the editors and the reviewers. Any product that may be evaluated in this article, or claim that may be made by its manufacturer, is not guaranteed or endorsed by the publisher.

## References

[1] Dubey JP Barr BC Barta JR Bjerkås I Björkman C Blagburn BL. Redescription of Neospora caninum and its differentiation from related coccidia. Int J Parasitol. (2002) 32:929–6. doi: 10.1016/S0020-7519(02)00094-2 12076623

[2] Reichel MP Alejandra Ayanegui-Alcérreca M Gondim LFP Ellis JT. What is the global economic impact of *Neospora caninum* in cattle - the billion dollar question. Int J Parasitol. (2013) 43:133–2. doi: 10.1016/j.ijpara.2012.10.022, PMID: 23246675

[3] Dubey JP Lindsay DS. Neosporosis, toxoplasmosis, and Sarcocystosis in ruminants. Vet Clin North Am Food Anim Pract. (2006). doi: 10.1016/j.cvfa.2006.08.001 17071358

[4] Dubey JP Lindsay DS Anderson ML Davis SW Shen SK. Induced transplacental transmission of Neospora caninum in cattle. J Am Vet Med Assoc. (1992) 201:709–3. PMID: 1399772

[5] Reichel MP Ellis JT. If control of Neospora caninum infection is technically feasible does it make economic sense? Vet Parasitol. (2006) 142:23–34. doi: 10.1016/j.vetpar.2006.06.027, PMID: 16893606

[6] Schock A Innes EA Yamane I Latham M Wastling J. Genetic and biological diversity among isolates of *Neospora caninum*. Parasitology. (2001) 123:13–23. doi: 10.1017/s003118200100796x 11467779

[7] Cabrera A Fresia P Berná L Silveira C Macías-Rioseco M Arevalo AP. Isolation and molecular characterization of four novel Neospora caninum strains. Parasitol Res. (2019) 118:3535–42. doi: 10.1007/s00436-019-06474-9, PMID: 31701296

[8] Chryssafidis AL Cantón G Chianini F Innes EA Madureira EH Gennari SM. Pathogenicity of Nc-Bahia and Nc-1 strains of *Neospora caninum* in experimentally infected cows and buffaloes in early pregnancy. Parasitol Res. (2014) 113:1521–8. doi: 10.1007/s00436-014-3796-x 24562816

[9] Khan A Fujita AW Randle N Regidor-Cerrillo J Shaik JS Shen K. Global selective sweep of a highly inbred genome of the cattle parasite Neospora caninum. Proc Natl Acad Sci U S A. (2019) 116:22764–73. doi: 10.1073/pnas.1913531116, PMID: 31636194 PMC6842595

[10] Rojo-Montejo S Collantes-Fernández E Blanco-Murcia J Rodríguez-Bertos A Risco-Castillo V Ortega-Mora LM. Experimental infection with a low virulence isolate of *Neospora caninum* at 70 days gestation in cattle did not result in foetopathy. Vet Res. (2009b) 40:49. doi: 10.1051/vetres/2009032, PMID: 19497257 PMC2704335

[11] Al-Qassab SE Reichel MP Ellis JT. On the biological and genetic diversity in *Neospora caninum*. Diversity. (2010) 2:411–8. doi: 10.3390/d2030411

[12] Aguado-Martínez A Basto AP Leitão A Hemphill A. Neospora caninum in non-pregnant and pregnant mouse models: Cross-talk between infection and immunity. Int J Parasitol. (2017) 47:723–5. doi: 10.1016/j.ijpara.2017.09.001 28903024

[13] Collantes-Fernández E Alvarez-García G Pérez-Pérez V Pereira-Bueno J Ortega-Mora LM. Characterization of pathology and parasite load in outbred and inbred mouse models of chronic Neospora caninum infection. J Parasitol. (2004) 90:579–3. doi: 10.1645/GE-3290, PMID: 15270102

[14] Dubey JP Hemphill A Calero-Bernal R Schares G. Neosporosis in animals. Florida, United States: CRC Press (2017).

[15] Hemphill A Aguado-Martínez A Müller J. Approaches for the vaccination and treatment of *Neospora caninum* infections in mice and ruminant models. Parasitology. (2016) 143:245–9. doi: 10.1017/S0031182015001596, PMID: 26626124

[16] Jia L Xie S Li J Li H Wang H Zhao S. Establishment of a model of *Neospora caninum* infection in pregnant mice. Parasitol Res. (2020) 119:3829–37. doi: 10.1007/s00436-020-06903-0, PMID: 33009944

[17] Piñeyro MD Parodi-Talice A Arcari T Robello C. Peroxiredoxins from Trypanosoma cruzi: virulence factors and drug targets for treatment of Chagas disease? Gene. (2008) 408:45–50. doi: 10.1016/j.gene.2007.10.014, PMID: 18022330

[18] Yamage M Flechtner O Gottstein B. *Neospora caninum*: specific oligonucleotide primers for the detection of brain “cyst” DNA of experimentally infected nude mice by the polymerase chain reaction (PCR). J Parasitol. (1996) 82:272. doi: 10.2307/3284160 8604096

[19] Faral-Tello P Greif G Romero S Cabrera A Oviedo C González T. *Trypanosoma cruzi* isolates naturally adapted to congenital transmission display a unique strategy of Transplacental passage. Microbiol Spectr. (2023) 11:e0250422. doi: 10.1128/spectrum.02504-22, PMID: 36786574 PMC10100920

[20] Berná L Rodriguez M Chiribao ML Parodi-Talice A Pita S Rijo G. Expanding an expanded genome: long-read sequencing of Trypanosoma cruzi. Microb Genom. (2018) 4:e000177. doi: 10.1099/mgen.0.000177, PMID: 29708484 PMC5994713

[21] Berná L Marquez P Cabrera A Greif G Francia ME Robello C. Reevaluation of the *toxoplasma gondii* and *Neospora caninum* genomes reveals misassembly, karyotype differences, and chromosomal rearrangements. Genome Res. (2021) 31:823–3. doi: 10.1101/gr.262832.120, PMID: 33906964 PMC8092007

[22] Li H Handsaker B Wysoker A Fennell T Ruan J Homer N. The sequence alignment/map format and SAMtools. Bioinformatics. (2009) 25:2078–9. doi: 10.1093/bioinformatics/btp352, PMID: 19505943 PMC2723002

[23] Danecek P Auton A Abecasis G Albers C Banks E DePristo MA. The variant call format and VCFtools. Bioinformatics. (2011) 27:2156–8. doi: 10.1093/bioinformatics/btr330, PMID: 21653522 PMC3137218

[24] Koboldt DC Zhang Q Larson DE Shen D McLellan MD Lin L. VarScan 2: somatic mutation and copy number alteration discovery in cancer by exome sequencing. Genome Res. (2012) 22:568–6. doi: 10.1101/gr.129684.111, PMID: 22300766 PMC3290792

[25] Robinson JT Thorvaldsdóttir H Winckler W Guttman M Lander ES Getz G. Integrative genome viewer. Nat Biotechnol. (2011) 29:24–6. doi: 10.1038/nbt.1754.Integrative, PMID: 21221095 PMC3346182

[26] Oliveros JC “VENNY. An Interactive Tool for Comparing Lists with Venn Diagrams. Http://Bioinfogp.Cnb.Csic.Es/Tools/Venny/Index.Html. Bioinfogp.Cnb.Csic.Es/Tools/Venny/Index.Html. 2007 (2007).

[27] Bjerkas I Jenkins MC Dubey JP. Identification and characterization of Neospora caninum tachyzoite antigens useful for diagnosis of neosporosis. Clin Diagn Lab Immunol. (1994) 1:214–1. doi: 10.1128/cdli.1.2.214-221.1994, PMID: 7496948 PMC368230

[28] Staubli D Nunez S Sager H Schares G Gottstein B. *Neospora caninum* immunoblotting improves serodiagnosis of bovine neosporosis. Parasitol Res. (2006) 99:648–8. doi: 10.1007/s00436-006-0207-y, PMID: 16718512

[29] Murillo-León M Müller B Zimmermann I Singh S Widdershooven P Campos C. Molecular mechanism for the control of virulent *toxoplasma gondii* infections in wild-derived mice. Nat Commun. (2019) 10. doi: 10.1038/s41467-019-09200-2, PMID: 30874554 PMC6420625

[30] Dellarupe A Regidor-Cerrillo J Jiménez-Ruiz E Schares G Unzaga JM Venturini MC. Clinical outcome and vertical transmission variability among canine Neospora caninum isolates in a pregnant mouse model of infection. Parasitology. (2014) 141:356–6. doi: 10.1017/S0031182013001479, PMID: 24148606

[31] Regidor-Cerrillo J Gómez-Bautista M Del Pozo I Jiménez-Ruiz E Aduriz G Ortega-Mora LM. Influence of *Neospora caninum* intra-specific variability in the outcome of infection in a pregnant BALB/c mouse model. Vet Res. (2010) 41:52. doi: 10.1051/vetres/2010024, PMID: 20416260 PMC2878169

[32] Amini L Namavari M Khodakaram-Tafti A Divar MR Hosseini SMH. The evaluation of attenuated *Neospora caninum* by long-term passages on murine macrophage cell line in prevention of vertical transmission in mice. Vet Parasitol. (2020) 283:109171. doi: 10.1016/j.vetpar.2020.109171, PMID: 32623187

[33] Jiménez-Ruiz E Álvarez-García G Aguado-Martínez A Ortega-Mora LM. Low rates of Neospora caninum infection reactivation during gestation are observed in both chronically and congenitally infected mice. Parasitology. (2013) 140:220–8. doi: 10.1017/S0031182012001515 23058003

[34] Quinn HE Miller CMD Ryce C Windsor PA Ellis JT. Characterization of an outbred pregnant mouse model of *Neospora caninum* infection. J Parasitol. (2002) 88:691–6. doi: 10.1645/0022-3395(2002)088[0691:COAOPM]2.0.CO;2, PMID: 12197115

[35] Rojo-Montejo S Collantes-Fernández E Regidor-Cerrillo J Álvarez-GarcÍa G Marugan-Hernández V Pedraza-Díaz S. Isolation and characterization of a bovine isolate of Neospora caninum with low virulence. Vet Parasitol. (2009a) 4:7–16. doi: 10.1016/j.vetpar.2008.10.009, PMID: 19027235

[36] Jiménez-Pelayo L García-Sánchez M Collantes-Fernández E Regidor-Cerrillo J Horcajo P Gutiérrez-Expósito D. Crosstalk between Neospora caninum and the bovine host at the maternal-foetal interface determines the outcome of infection. Vet Res. (2020) 51:83. doi: 10.1186/s13567-020-00803-y, PMID: 32552750 PMC7302351

[37] Hehl A Krieger T Boothroyd JC. Identification and characterization of SRS1, a toxoplasma gondii surface antigen upstream of and related to SAG1. Mol Biochem Parasitol. (1997) 89:271–2. doi: 10.1016/s0166-6851(97)00126-6, PMID: 9364971

[38] Risco-Castillo V Marugán-Hernández V Fernández-García A Aguado-Martínez A Jiménez-Ruiz E Rodríguez-Marco S. Identification of a gene cluster for cell-surface genes of the SRS superfamily in Neospora caninum and characterization of the novel SRS9 gene. Parasitology. (2011) 138:1832–42. doi: 10.1017/S0031182011001351, PMID: 21939586

[39] Mills EL Kelly B O'Neill LAJ. Mitochondria are the powerhouses of immunity. Nat Immunol. (2017) 18:488–8. doi: 10.1038/ni.3704, PMID: 28418387

[40] Riley JS Tait SW. Mitochondrial DNA in inflammation and immunity. EMBO Rep. (2020) 21:e49799. doi: 10.15252/embr.201949799, PMID: 32202065 PMC7132203

[41] Li X Straub J Medeiros TC Mehra C den Brave F Peker E. Mitochondria shed their outer membrane in response to infection-induced stress. Science. (2022) 375. doi: 10.1126/science.abi4343 35025629

[42] Zhang H Liu J Ying Z Li S Wu Y Liu Q. Toxoplasma gondii UBL-UBA shuttle proteins contribute to the degradation of ubiquitinylated proteins and are important for synchronous cell division and virulence. FASEB J. (2020) 34:13711–25. doi: 10.1096/fj.202000759RR 32808330

[43] Calarco L Barratt J Ellis J. Genome wide identification of mutational hotspots in the apicomplexan parasite *Neospora caninum* and the implications for virulence. Genome Biol Evol. (2018) 10:2417–31. doi: 10.1093/gbe/evy188, PMID: 30165699 PMC6147731

[44] Calarco L Ellis J. Contribution of introns to the species diversity associated with the apicomplexan parasite, Neospora caninum. Parasitol Res. (2020) 119:431–5. doi: 10.1007/s00436-019-06561-x 31901106

